# Fc Engineering Strategies to Advance IgA Antibodies as Therapeutic Agents

**DOI:** 10.3390/antib9040070

**Published:** 2020-12-15

**Authors:** Geert van Tetering, Mitchell Evers, Chilam Chan, Marjolein Stip, Jeanette Leusen

**Affiliations:** 1 Center for Translational Immunology, UMC Utrecht, Heidelberglaan 100, 3584 CX Utrecht, The Netherlands; g.j.vantetering@umcutrecht.nl (G.v.T.); j.g.m.evers-8@umcutrecht.nl (M.E.); c.l.chan@umcutrecht.nl (C.C.); m.c.stip@umcutrecht.nl (M.S.); 2 TigaTx BV, Yalelaan 40, 3584 CM Utrecht, The Netherlands

**Keywords:** IgA, engineering, glycosylation, half-life, FcαRI, FcRn, IgG, neutrophil

## Abstract

In the past three decades, a great interest has arisen in the use of immunoglobulins as therapeutic agents. In particular, since the approval of the first monoclonal antibody Rituximab for B cell malignancies, the progress in the antibody-related therapeutic agents has been incremental. Therapeutic antibodies can be applied in a variety of diseases, ranging from cancer to autoimmunity and allergy. All current therapeutic monoclonal antibodies used in the clinic are of the IgG isotype. IgG antibodies can induce the killing of cancer cells by growth inhibition, apoptosis induction, complement activation (CDC) or antibody-dependent cellular cytotoxicity (ADCC) by NK cells, antibody-dependent cellular phagocytosis (ADCP) by monocytes/macrophages, or trogoptosis by granulocytes. To enhance these effector mechanisms of IgG, protein and glyco-engineering has been successfully applied. As an alternative to IgG, antibodies of the IgA isotype have been shown to be very effective in tumor eradication. Using the IgA-specific receptor FcαRI expressed on myeloid cells, IgA antibodies show superior tumor-killing compared to IgG when granulocytes are employed. However, reasons why IgA has not been introduced in the clinic yet can be found in the intrinsic properties of IgA posing several technical limitations: (1) IgA is challenging to produce and purify, (2) IgA shows a very heterogeneous glycosylation profile, and (3) IgA has a relatively short serum half-life. Next to the technical challenges, pre-clinical evaluation of IgA efficacy in vivo is not straightforward as mice do not naturally express the FcαR. Here, we provide a concise overview of the latest insights in these engineering strategies overcoming technical limitations of IgA as a therapeutic antibody: developability, heterogeneity, and short half-life. In addition, alternative approaches using IgA/IgG hybrid and FcαR-engagers and the impact of engineering on the clinical application of IgA will be discussed.

## 1. Introduction: IgA and FcαRI

**Figure 1:**
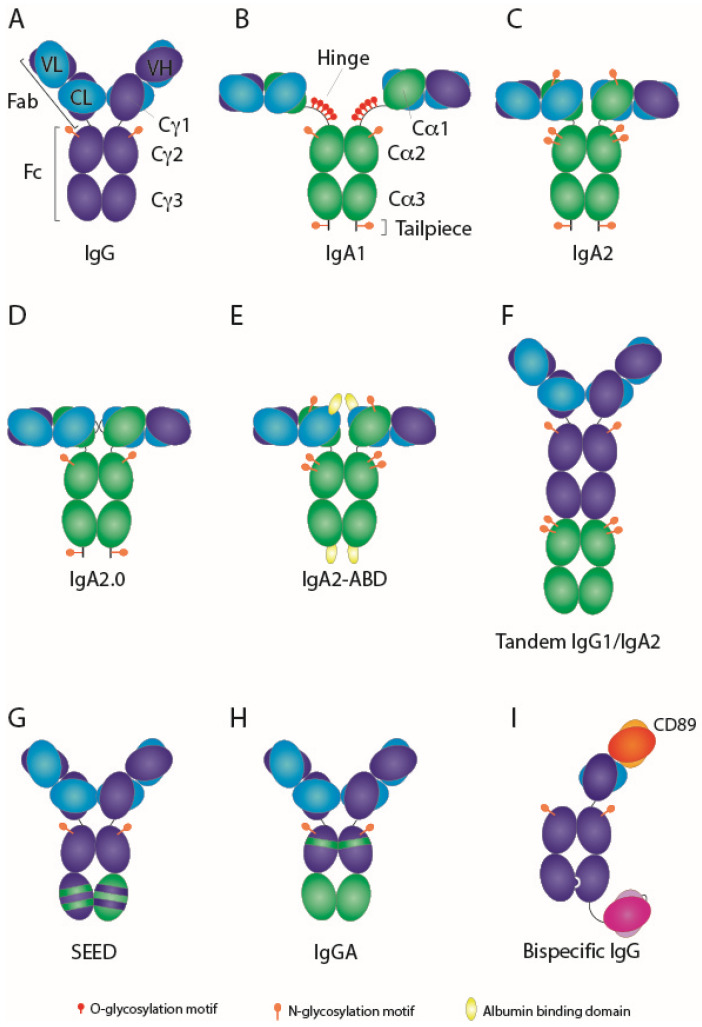
Schematic overview of IgG and IgA formats. (**A**) IgG1 is depicted, with heavy chain in dark blue and light chain in light blue. The variable domains of IgG have been grafted onto IgA creating (**B**) a chimeric IgA1, with heavy chain in green, or (**C**) chimeric IgA2m (1). (**D**) Engineering IgA2m (1) to improve stability and homogeneity resulted in IgA2.0 [26]. (**E**) Albumin-binding domain (ABD) in yellow fused to IgA2m (1) [27]. Illustration of hybrid IgG-IgA molecules: (**F**) Tandem IgG1-IgA [52], (**G**) strand-exchange engineered domain (SEED)-bodies [53], and (**H**) Cross-isotype IgGA [54]. (**I**) FcαRI-engaging (red/orange) × CD20 (pink/purple) bispecific antibody [55].

Antibodies in humans can be classified into five isotypes, IgM, IgD, IgE, IgG, and IgA. IgG antibodies are the most prevalent isotype in circulation, accounting for about 70–80%, followed by IgA, making up 15–20% of serum immunoglobulins. At the mucosal surface, IgA is the most abundant antibody, where it acts in neutralizing pathogens [1]. About 75% of the total antibody production constitutes of IgA, making it the most produced antibody in the human body [2]. IgA consists of two subclasses, IgA1 and IgA2, showing a high sequence identity of around 90%. IgA1 and IgA2 structurally differ in a 13-residue longer hinge fragment of IgA1, which contains several *O*-linked glycosylation sites (Figure 1). Three allotypes of IgA2 have been described, IgA2m (1), IgA2m (2), and IgA2(n) [3], of which the first two have been described in detail and as such will be discussed here. Where IgA1 harbors 2 *N*-linked glycosylation motifs, IgA2m (1) and IgA2m (2) contain 4 or 5 *N*-linked glycosylation sites, respectively. Whereas most of the isotypes can be found in only one configuration, IgA has been shown to be highly versatile, existing as polymeric, dimeric, and secretory IgA at mucosal tissues. In serum, IgA is predominantly found as a monomer, where IgA1 makes up to 90% of the monomeric IgA fraction found in blood [4]. Dimeric IgA is composed of two monomers joined together by a J-chain and plays a role in mucosal immunity protecting the host against harmful pathogens [5]. Dimeric IgA primarily associates with the polymeric Ig receptor (pIgR) expressed on epithelial cells, allowing dimeric IgA to be transcytosed across the epithelium to the luminal side [6]. The majority of the extracellular domain of pIgR associated with dimeric IgA is cleaved by unknown mechanisms and remains attached to the dimer acting as the secretory component resulting in secretory IgA. The secretory component provides protection from enzymatic breakdown in mucosal areas [7]. As a monomer, IgA interacts with serum proteins where it likely plays an anti-inflammatory role [8]. I, n mice that have been stimulated with G-CSF IgA is able to opsonize bacteria in circulation to be phagocytosed by Kupffer cells [9] but IgA also displays anti-inflammatory functions by inhibiting pro-inflammatory responses in monocytes [10].

A set of receptors have been described to bind IgA antibodies in their multiple forms, either binding the Fc tail, carbohydrate side chains, J-chain, or secretory component. These comprise amongst others of pIgR, FcαRI (CD89), Fcα/µR, asialoglycoprotein, and transferrin receptors. The main receptor binding monomeric IgA antibodies is the low-affinity Fc receptor FcαRI (*K*a = ~10^6^ M^−1^), which interacts with IgA via its Cα1 and Cα2 domains, primarily after antibody–antigen recognition. FcαRI is able to bind both monomeric and dimeric IgA, but binding of secretory IgA is largely impeded due to steric hindrance by the secretory component [11,12]. FcαRI is a 55–75 kDa type I transmembrane receptor composed of two extracellular Ig-like domains, a transmembrane domain, and a cytoplasmic tail. It is expressed on myeloid cells such as neutrophils, eosinophils, monocytes, subsets of dendritic cells, and macrophages [13]. Soluble monomeric IgA only transiently interacts with the receptor due to low affinity but can stably interact with high avidity once complexed, thereby crosslinking the receptor [14,15]. FcαRI engagement with IgA-opsonized targets or immune complexes induces signaling via its associated FcR γ-chain bearing ITAM motifs, which become phosphorylated, allowing docking of cellular proteins such as Syk and Src family protein tyrosine kinases that on their end induce a signaling cascade. This increases intracellular levels of free Ca^2+^ and causes a series of cellular functions such as antibody-dependent cellular cytotoxicity (ADCC), phagocytosis, antigen presentation, endocytosis, superoxide generation, inflammatory mediator release, and cytokine production. However, also anti-inflammatory roles have been attributed to monomeric IgA binding through the recruitment of SHP-1 by an inhibitory ITAM configuration [10,16]. In the remainder of this review, we will focus mostly on monomeric IgA since, in our view, it has the best format to be employed as a systemically active therapeutic antibody.

A first demonstration of the therapeutic potential of IgA against tumor targets has been given by Valerius et al., showing ADCC by IgA antibodies via FcαRI-expressing polymorphonuclear leukocytes (PMNs) [17]. Early examples of recombinant (chimeric) IgA monomeric antibodies were made some years later, assessing the effect of IgA on tumor cell lines resulting in effective ADCC [18,19,20]. The development of FcαRI transgenic mouse models [21,22] greatly improved possibilities for pre-clinical studies and enabled the first in vivo study of the therapeutic effect of IgA on tumors [23]. In a direct comparison with its IgG1 counterpart, an IgA2-EGFR showed similar or better anti-tumor responses in SCID xenograft or immunocompetent models demonstrating the potential of IgA in a therapeutic setting. Nevertheless, IgA serum levels remained significantly lower than IgG despite a multiple dosing regimen, showing about 5-fold lower levels than IgG1. This suggests an underestimation of the true capacity of IgA to target cancer. More studies followed, showing the potential of recombinant IgA antibodies as an effective immunotherapy agent targeting cancer in vivo [24,25,26,27,28].

Furthermore, Brandsma et al. demonstrated that IgA induces strong crosslinking of the FcαRI in neutrophils after target recognition, activating FcαRI in a bivalent fashion, thereby activating two associated FcR γ-chains per receptor. Thus, per IgA molecule in total four ITAMs will become activated, resulting in a potent downstream signal leading to a more pronounced effect, often stronger than with IgG [29,30,31,32]. Next to this, the potential of IgG is strongly determined by the expressed Fc-gamma receptors. Effector mechanisms of IgG1 are mediated by NK cells in particular, via their FcγRIIIa [33]. Macrophages, however, express one high-affinity activating, two low-affinity activating, and one inhibitory Fcγ receptor [34], alongside a FcαRI. On neutrophils, FcγRIIa is expressed twice as high as FcαRI, suggesting that IgG1 should induce higher activity. Yet, we and others have demonstrated that next to FcγRIIa also the decoy receptor FcγRIIIb is expressed in high levels, scavenging IgG1 antibodies, thereby explaining the lower potential of IgG1 [35,36]. However, if FcγRIIIb is blocked, the IgG1 is still not able to induce cytotoxicity to the level of IgA. One possible explanation for this is that FcγRIIa contains a single ITAM within its intracellular domain that becomes activated upon IgG1 binding and starts to signal as briefly described above. As a consequence of the described mechanism and taken into account the fact that neutrophils are present in high numbers in blood, IgA shows promise to act as a highly potent antibody.

Despite its success in pre-clinical models, no clinical studies employing monomeric IgA antibodies targeting cancer have been started yet. This has been due to a lack of pre-clinical models investigating the therapeutic efficacy of IgA in animals, as mice do not express the FcαRI. This has been greatly improved by the transgenic expression of human FcαRI in mice [21,22]. This model closely resembles the human distribution of FcαRI in blood, showing expression on neutrophils, eosinophils, and activated tissue-resident macrophages, however, circulating monocytes do not express the receptor. Technical limitations of IgA manufacturing still withhold IgA from entering clinical studies, which can be classified into three categories that will be discussed below in detail: (1) issues in recombinant IgA production and subsequent purification processes due to aggregate formation, (2) heterogeneity in oligosaccharide content of IgA because of multiple *N*-glycosylation motifs, (3) short serum half-life of IgA. High-level antibody engineering strategies to overcome these technical limitations of IgA, including the development of IgA–IgG hybrid molecules and FcαRI engagers, have been implemented and are still ongoing. This review will detail the most recent developments in IgA engineering that might eventually open up the road to the clinic.

## 2. Enhancing IgA Developability

Out of the three options, IgA2m (1) seems the most attractive subtype to develop as a therapeutic antibody since IgA2m (2) has an additional *N*-glycosylation motif, whereas IgA1 has next to *N*- also *O*-glycosylation that is associated with Berger disease [37]. However, although both IgA1 and IgA2m (2) are able to covalently link heavy and light chain via disulfide bonds, regulating their stability, IgA2m (1) lacks this feature [38]. To improve the stability of IgA2m (1), we implemented a series of modifications. First, to facilitate covalent linkage between heavy and light chain, a P221R mutation has been introduced mirroring the exact same residues found in IgA2m (2) that sterically allows the formation of an alternate disulfide bond between the heavy (C241) and the light chain cysteines (C214) [39]. This resulted in reduced free kappa light chain production and improved monomeric thermal stability. This mutated IgA is still able to retain its full efficacy in ADCC by PMN.

Isotype switching does not seem to compromise its production rates in human production systems, as both IgG and IgA isotypes of Trastuzumab and Pertuzumab were shown to have similar yields [40]. However, there have been conflicting studies concerning the effects of isotype switching, using identical variable domains, on antigen binding. As we generally see no or minor differences in *K*_D_ values for IgG1, IgA1, and IgA2 variants, i.e., anti-CD20 [41], anti-EGFR [18], anti-HER2 [40], and anti-GD2 antibodies (Evers et al. submitted), others observe major differences. For instance, antigen-binding affinity towards tubulin was significantly higher for an IgA1 isotype as compared to IgG1 [42]. This difference was associated with the CH1 domain, which was revealed in high-resolution crystal structures to be more rigid than its IgG1 counterpart. Indeed, the IgA1 hinge is more rigid than the typical IgG1 hinge [43,44]. Rigidity in the hinge region promotes long-distance allosteric signaling from the V-region to the C-region and subsequently alters CH conformation [45,46].

Unlike IgG antibodies, IgA antibodies (Figure 1) do not harbor a protein A binding site, making it impossible to purify IgA antibodies using existing IgG-based methods (i.e., recombinant protein A resins). Although several methods have been described to capture IgA, they all have their limitations. For instance, using the lectin Jacalin, which specifically binds *O*-glycosylated proteins, solely the IgA1 isotype can be isolated [47]. Protein L only binds to certain framework sequences of specific kappa light chain families [48], although some studies showed that this could be overcome by introducing small mutations in the light chain [49,50]. We applied pharmaceutical principles on the production of recombinant IgA antibodies, using the pharmaceutical type of vectors in a serum-free CHO system, obtaining yields similar to IgG antibodies [51]. Employing a multi-step procedure implementing kappa light chain capture followed by size exclusion chromatography to isolate monomeric antibodies, recombinant IgA could be successfully isolated [39,51]. In addition, current Fab-based IgA-specific resins such as CaptureSelect are efficient in isolating IgA antibodies to high purity as well.

By design of nature, IgA antibodies contain an α-tail that is used in the formation of di- or oligomers. To form multimers, typically a J-chain is required; however, also in the absence of the J-chain, multimeric IgA can be formed [51]. This poses a manufacturability issue and, therefore, engineering has been employed to prevent polymer formation. In the IgA2m (1) molecule, we removed two C-terminal residues from the tailpiece, which are crucial for IgA dimer formation [56]. Likewise, monomeric IgA in serum has lost its most C-terminal Tyr residue, possibly impacting its stability as a monomer, yet no clear biological relevance has been found [57]. Whilst antibody monomerization was increased, Fab- and Fc-mediated effector functions were maintained. To reduce the formation of dimeric aggregates and complex formation with serum proteins, two free cysteines were substituted [26]. Interestingly, a single substitution of C320S present in the CH1 region of IgA2m (2) containing a wild type tailpiece resulted in a highly stable mainly monomeric IgA molecule [28]. Additional substitution of the C480S or complete deletion of the tailpiece shows an even better profile, with no high molecular weight species present at all. However, this seems to contrast previous results implicating poor assembly of IgA1 due to either loss of the tailpiece or by substituting the penultimate Cys residue, which resulted in the formation of half-molecules consisting of a paired single heavy and single light chain only [58]. Most likely, an additional Cys mutation in CH2 as described for IgA2m (2) might solve the issue for IgA1 [28].

Modifying critical residues in the IgA molecules solves major developability issues, resulting in more stabilized IgA2m (1) molecules that have an intrinsic lower tendency to aggregate. This clearly eases up antibody purification processes, resulting in a clean and pure IgA protein.

## 3. IgA Glyco-Engineering

Measurements of human IgA in the circulation of hFcαRI transgenic SCID mice indicate a half-life of only 15 h, whereas IgG has a half-life of at least 4 days [23]. However, the serum half-life of IgG is about 21 days, while IgA has an estimated half-life in circulation between 4 and 7 days in humans and other primates [59,60,61]. Although half-life studies in mice are informative, they cannot be translated to humans directly. Cross-species differences in affinity of hIgG for mouse or human FcRn makes it difficult to properly interpret half-lives [62,63]. Moreover, half-life also depends on the type of mouse model employed [64,65].

With only a single *N*-linked glycosylation site in IgG and 2 or 4–5 *N*-linked glycosylation sites in IgA1 and IgA2, respectively, IgA has a more heterogeneous *N*-glycosylation pattern [66], partly attributing to a shorter half-life of IgA. In particular, free galactoses are highly susceptible to clearance by the asialoglycoprotein receptor (ASGPR), as genetic disruption or blockade of this receptor significantly improves IgA half-life [23,67]. Moreover, terminal mannoses are susceptible to a different type of scavenging receptor, the mannose receptor [68,69]. A fully sialylated IgA molecule would be less affected by ASGPR clearance, thus increasing its serum half-life. We showed that complementing production cell lines with the enzymes responsible for the addition of terminal sialic acids to the antibodies could extend their serum exposure time [28]. However, the addition of glycan precursors to the medium does not reach physiological glycosylation levels of antibodies, where 64% of serum IgA1 is sialylated [28,70].

Differences in glycosylation of IgA1 and IgA2 impact their function, inducing different signaling in neutrophils [4]. By nature, monomeric IgA1 antibodies have a higher content of terminal sialic acids, whereas IgA2 is less sialylated while having more glycans per protein. Upon enzymatic removal of sialylation of IgA1, its effector functions more closely resemble that of IgA2, showing pro-inflammatory effects [4]. This demonstrates *N*-glycosylation status on IgA antibodies is critical for their function.

To simplify glycosylation and thereby improve drug homogeneity, two *N*-glycosylation motifs in IgA2m (1) were removed by amino acid substitution. These amino acids were replaced by the corresponding IgA1 counterparts, which resulted in, amongst other mutations, the IgA2.0 antibody, only harboring two *N*-glycosylation motifs [26]. Implementing mutations in the antibody’s constant region may have consequences for the antigen-binding affinity and *vice versa*. FcαRI binding to the IgA CH2-CH3 domain induces conformational changes at the distal hinge and Fab region [46]. Moreover, mutating two critical residues in the CH3 domain of IgA1 (C266/H317) or IgA2 (C253/H304) hampered signaling through the hinge to the variable domain and consequently lowered the antigen-binding affinity [71]. Likewise, in the other direction, antigen-binding causes conformational changes in the Fc domain [72]. However, the mutations described here to generate IgA2.0 eased the developability and improved stability without negatively affecting antigen-binding or effector functions.

The loss of *N*-glycosylation sites did not have any consequences for the EGFR-directed Fab- or Fc-mediated effector functions. Moreover, it even showed improved characteristics in terms of production and purification. The IgA2.0 antibody displayed significantly improved half-life in SCID mice. The typical drop in antibody concentration within the distribution phase observed for IgA2m (1) was less in IgA2.0, exhibiting less susceptibility to initial ASGPR-induced clearance [26].

Glycosylation in polymeric IgA, consisting of two or more IgA molecules and the joining chain, is even more heterogeneous. Mono-, di-, or tetramers show reduced sialylation levels compared to monomeric human serum IgA, which shows a level of over 90% sialylation [73]. Of note, the joining chain also contributes to these glycosylation levels. As a result of the high sialylation levels of serum IgA, its half-life is significantly better than recombinantly produced IgA, either mono- or dimeric. Assessing site-specific glycosylation of the *N*-glycosylation motif present in the tailpiece reveals no glycan is present in ±40% of the cases. Strikingly, by mutating this same glycosylation site, the balance was favored toward increased polymer formation [73]. This shows that glycosylation partially dictates the formation of IgA multimers.

By engineering a fully aglycosylated IgA2m (2) polymeric antibody, the effect of the scavenging receptors on the half-life could be properly investigated. Although the aglycosylated IgA polymer showed the highest level of transcytosis in vitro, this did only result in a relatively small increase in half-life, particularly within the distribution phase [73].

Another approach to arrange different glycosylation patterns is to produce IgA in plants. Production of monomeric IgA in *Nicotiana benthamiana* plants resulted in antibodies mainly carrying complex-type biantennary *N*-glycans, as opposed to HEK293-produced IgA showing a much more heterogeneous *N*-glycosylation pattern [74,75]. By producing IgA1, IgA2m (1), and IgA2m (2) in a plant-based system in the presence or absence of critical enzymes for *N*-glycosylation, its effect on antibody characteristics could be assessed [76]. *N*-glycan modifications affected the thermal stability of the antibodies, but not Fab-mediated binding to HER2. In addition, the glycosylation status of the FcαRI was assessed in combination with the altered glycosylation variants of IgA. In contrast to the IgA *N*-glycans reported earlier [77], FcαRI *N*-glycans significantly contributed to the binding interaction showing higher affinity upon simpler (i.e., oligomannosidic- or single GlcNac) glycosylation of the receptor. Whereas FcαRI normally has the highest affinity for IgA1 (*K*_D_ = ~155 nM), diminishing FcαRI glycosylation resulted in higher affinity levels for IgA2, getting in the IgA1 affinity range. Moreover, fully aglycosylated FcαRI resulted in equal but ∼6-fold higher affinity to all three IgA iso/allotypes tested (*K*_D_ = ~20 nM) [76].

Glyco-engineering of IgA antibodies by removing *N*-glycosylation motifs does result in a longer half-life in the alpha phase of metabolism. Yet, this effect does not seem to be fully mediated by ASGPR clearance, as fully aglycosylated polymeric IgA antibodies show slightly elevated half-lives but not similar to IgG. However, as this concerns dimeric IgA, it would be interesting to further glyco-engineer and assess the half-lives of monomeric IgA molecules. Plant-based production would allow for specific *N*-glycosylation patterns, but plant-produced *N*-glycans are less sialylated in general, which will negatively affect the half-life of IgA.

## 4. FcRn-Based Half-Life Extension Strategies

Although early clearance of monomeric IgA might be largely attributed to the ASGPR, other mechanisms clear antibodies rapidly after distribution. A critical mechanism regulating antibody half-life is the neonatal receptor FcRn. In early life, FcRn is responsible for the passage of maternal antibodies to fetuses via the placenta [78,79], but throughout life, this receptor is also important for recycling or transcytosis of proteins such as IgG antibodies and albumin [80,81,82,83]. In the case of IgG, interaction with FcRn rescues the immunoglobulin from intracellular degradation through a cellular recycling mechanism [84]. Upon cellular uptake and subsequent vesicular trafficking, IgG ends up in acidic endosomes, where it is able to bind FcRn due to the low pH. Subsequent exocytosis exposes the IgG-FcRn complex to physiological pH, thereby releasing IgG from FcRn into the extracellular milieu. IgG molecules, such as IgG3 antibodies that are unable to bind FcRn in the sorting endosomes, are subject to degradation. This intracellular recycling process has a major impact on the persistence of IgG antibodies, increasing their serum half-life significantly [85,86]. Whereas IgG exhibits FcRn binding to protect itself against catabolism, IgA does not have this binding capacity as it lacks an FcRn binding site, thereby severely reducing its half-life, due to degradation upon cellular uptake.

To investigate whether FcRn binding would improve IgA half-life, several approaches have been undertaken to introduce binding to FcRn. To take advantage of the FcRn-mediated recycling of albumin, an albumin-binding domain (ABD) has been coupled to IgA, either linked to the light- or heavy chain [27]. These antibodies show binding to albumin of different species and still retain Fab-mediated target binding and Fc-mediated binding to the FcαRI. ABD-coupled antibodies do show an increase in half-life by two days compared to unmodified IgA molecules when injected in mice, regardless of administration route. Thus, IgA-ABD-albumin binding to FcRn and subsequent recycling occurs in vivo. Since the ABD replaces the tailpiece of IgA2 harboring an *N*-glycosylation motif, ASGPR-mediated clearance is possibly diminished as well. Despite their longer half-life in vivo, ADCC was impaired in the presence of albumin, regardless of where ABD was incorporated. Strikingly, however, therapeutic efficacy in xenograft models was slightly improved. Overall serum levels of IgA were higher, likely accounting for this observed therapeutic effect [27]. Because of the low affinity of mouse albumin for mouse FcRn, the potential of this approach is underestimated and could be properly addressed in human FcRn Tg mice (without expression of mouse serum albumin).

Although ABD coupling to IgA has a positive effect on the half-life, it also negatively affects ADCC in some cases. To be developed further, some improvements need to be made, e.g., by using a less bulky molecule that does not impact ADCC functions, by the introduction of FcRn binding site into the Fc. However, the latter would likely interfere with the FcαRI binding and needs careful consideration if implemented.

## 5. IgA-IgG Hybrid Molecules

Several other approaches take advantage of FcRn binding of IgG by fusion of IgG and IgA molecules into a single hybrid molecule [52,55,73]. In one approach, the Cα2-Cα3 Ig-domains were coupled to the IgG1 C-terminal end, creating an IgG1/IgA2 hybrid molecule directed against HER2 [52]. The hybrid protein retained receptor binding for the different FcγRs, FcRn, C1q, and FcαRI. Therefore, ADCC using the hybrid protein with either IgG1 cognate effector cells (NK) or IgA cognate effector cells (PMN) both contributed to cell killing and the combination of PMN and PBMC led to a small but consistent gain in ADCC activity. There was only a small additive effect, which could be attributed to the fact that the hybrid antibody recruits two different effector cells on the same target, where probably the antigen is limiting. Similarly, combining separate IgA2 and IgG1 antibodies targeting the same antigen does not augment killing [31], as the antigen is the limiting factor here. Hybrid IgG1/IgA2 showed similar phagocytosis compared to single HER2 antibodies. Moreover, the antibody serum half-life of the IgG1/IgA2 hybrid protein exhibited a similar clearance rate as the single IgG1 molecule [52], which has been confirmed in a later study using the same hybrid molecule as a CD20-targeting antibody [55].

The CD20-IgG1/IgA2 outperformed single agents IgG1- and IgA2-CD20 in tumor cell killing in vitro using mouse effector cell populations. Moreover, using bone marrow-derived macrophages, a similar amount of phagocytosis was observed for IgG1/IgA2 hybrids and IgA2-CD20 antibodies, whereas IgG1-CD20 was less effective in ADCP. To study the CD20-hybrid molecule in vivo, a FcαRI transgenic mouse, where FcαRI expression is restricted to monocytes and macrophages, has been used. When FcαRI transgenic or wild-type mice with human CD20 expressing cells were treated with these antibodies, no effect of IgA2-CD20 was observed in wild-type mice, whereas the hybrid protein performed as well as its IgG1 counterpart. In FcαRI transgenic mice, the IgG1/IgA2-CD20 molecule showed improved tumor regression in comparison to IgG1- or IgA2-CD20 alone, which was mediated by macrophages [55].

In contrast to full IgA-domain addition to IgG, several studies investigated grafting structurally related and conserved sequences of IgA in the Cγ3 domains to combine the functions of IgG and IgA to develop a bispecific antibody platform. This is termed strand-exchange engineered domain (SEED) [53]. This strategy creates two asymmetric CH3 domains by alternating contacting residues from each immunoglobulin, having modified β-sheet domain interfaces, each of which were termed AG and GA. This design disfavored homodimer formation and due to the distinct complementary contact surfaces between AG and GA monomers, efficient heterodimer generation was achieved. Next to that, SEED bodies retained binding to recombinant protein A and FcRn, easing purification processes and granting long half-life. To functionally assess the potential of SEED bodies, the clinically validated C225 antibody against EGFR was reformatted into mono- and dimeric SEED body [87]. Competitive radioligand binding assays showed that SEED body variants were able to inhibit EGF binding to its receptor, blocking EGFR. SEED bodies induced similar cytotoxicity as control 225-IgG antibodies in PBMC-ADCC targeting A431 cells. For CDC a hu14.18 variable domain was grafted onto the SEED body and complement-induced lysis was measured on M21 melanoma cells, resulting in somewhat lower CDC than fully IgG-Fc. The pharmacokinetics of SEED bodies assessed in nude mice demonstrated half-life is similar to their IgG counterparts. However, thermal stability was slightly reduced. The main applicability of SEED bodies is as heterodimeric scaffolds for a variety of binding moieties, such as scFv and different Fab domains [87,88]. Unfortunately, it has not been addressed whether SEED bodies are able to bind to FcαRI.

A similar approach which combined functions of IgA and IgG into a single molecule was described by Chintalacharuvu et al. Here, the addition of Cα3 to the C-terminus of the IgG1 Fc portion (γγγα), and/or substitution of Cγ1 for Cα1 (αγγα), shows better stability to extreme pH conditions, which negatively impacts their serum half-life as dissociation from FcRn fails [89]. In addition, biosensor assays indicated that the avidity for FcRn was much higher for the hybrid IgG1/IgA antibodies than IgG1 only. This was especially true for the γγγα hybrid. However, whereas IgG dissociates at pH 7.4, the dissociation from FcRn was worse for the hybrid antibodies at physiological pH, possibly due to polymer formation due to the remaining α-tail in the Cα3 domain. These polymers would allow for more FcRn-binding sites. Because dissociation from FcRn does not occur at physiological pH, this molecule showed similar half-life values as wild type IgA2.

Another IgG-IgA hybrid molecule combining critical domains from IgA engrafted into IgG has been described. An in-depth Alanine-scanning analysis determined which residues were essential for FcαRI binding [54]. Out of several variants, a single IgG-IgA hybrid antibody displayed proper assembly and demonstrated both binding to FcαRI and certain FcγRs. In this cross-type hybrid, termed IgGA, the residues from the α1 loop of IgG-CH2 were replaced by respective IgA counterpart residues plus an additional glycine to match the length of IgG. Furthermore, the full CH3 domain was derived from IgA, replacing the IgG-CH3. An IgGA with the trastuzumab Fab region was engineered and tested in cytotoxic assays. The amount of ADCC by neutrophils induced by IgGA is similar to that of IgA, indicating the FcαRI is engaged and functionally utilized. In a phagocytosis assay using macrophages, IgG was outperformed by IgGA but showed similar levels of ADCP as IgA. This again shows this cross-isotype antibody is capable of activating the FcαRI. To assess the IgG effector functions, a complement activation assay was performed, showing that in contrast to wild-type IgA, the C1q-binding site was retained in the IgGA molecule as it is capable of inducing CDC. This shows effector functions of IgA have been implemented successfully into an IgG molecule, engaging similar effector cell populations as IgA, and enabling the induction of CDC. However, it remains to be determined if IgGA is capable of inducing ADCC using PBMC or total leukocyte fractions. In addition, no FcRn binding could be determined possibly resulting in a relatively short half-life. Unfortunately, no in vivo models have been used to address how this molecule would behave in physiological conditions. Yet, the use of two populations of effector cells on the same target does not seem to improve the cytotoxic effect [31].

## 6. FcαRI Engagers as IgA Alternative

A different approach using an IgG-based molecule engaging neutrophils for killing tumor targets has been described in the late 1990s [17,29]. This chemically engineered bispecific IgG F(ab’)_2_ targets both FcαRI and HER2. This antibody is able to recruit neutrophils and monocytes to target cells for ADCC or phagocytosis. Combining FcαRI and HER2 or EGFR targeting specificities, efficient killing by ADCC could be achieved using PMN, monocytes, and whole blood as effector cells [29]. Furthermore, using the same F(ab’)_2_ format targeting CD20 proved to be very efficient using a FcαRI x CD20 bispecific antibody, inducing lysis in a panel of malignant B-cells, which could not be achieved by either FcγR-targeting bispecific molecules or single CD20-targeting antibodies [90]. When assessing FcγRI x HER2 targeting F(ab’)_2_ bispecifics using monocyte-derived macrophages, the efficacy of FcγR mediated lysis could be enhanced by cytokines such as IFN-γ, in contrast to FcαRI x HER2/neu. However, stimulating monocyte-derived macrophages with myeloid growth factors significantly enhanced FcαRI-mediated lysis, where FcαRI-targeting F(ab’)_2_ bispecific antibodies even further increased lysis as compared to their FcγR counterparts [30,90]. Yet, none of these above-mentioned FcαRI-targeting bispecific molecules have been described in vivo.

In a more recent approach using a knobs-into-holes one-arm bispecific heterodimer, one arm consists of a CH2-CH3 domain only, pairing with a regular antibody arm composed of an N-terminal Fv-targeting FcαRI but carrying a CD20-targeting scFv at its C-terminal Fc-portion [91]. The in vivo efficacy has been assessed using injected activated human PMN in tumor-bearing SCID mice, demonstrating a clear reduction in tumor growth upon FcαRI x CD20 bispecific antibody administration. Furthermore, upon administering an Fc-silent version of the bispecific molecule into FcαRI transgenic mice expressing FcαRI on monocytes and macrophages only, tumor progression was significantly reduced, even outperforming the anti-CD20 antibody Rituximab [91].

However, there are some intrinsic limitations to this approach, since using a fully functional Fc portion could result in the bilateral killing of effector cells by ADCC. Therefore, it is key to test this type of molecules in a physiological setting to investigate this kind of fratricide. On the other hand, immune cells may employ several mechanisms preventing intercellular cytotoxicity of effector cells, using complement regulatory proteins avoiding complement activation [92,93], whereas the CD47-SIRPα axis might act as a barrier preventing undesired antibody-mediated cytotoxicity on healthy tissue including effector cells [94]. Of note, it is to be expected that the half-life of bispecific antibodies engaging neutrophils with high-affinity FcαRI antibodies is dramatically reduced, as this would probably result in a ‘sink’ effect after administration of directly binding the FcαRI receptor, thereby limiting efficacy. As neutrophils in the blood are short-lived [95], this would argue for a swift clearance of antibodies. Lastly, the mechanism of FcαRI activation by FcαRI-targeting bispecific antibodies would differ from natural activation, as single-arm FcαRI-targeting molecules do not have the potential of clustering FcαRI, which IgA antibodies are capable of [32]. Therefore, prior to entering the clinical phase with FcαRI-targeting bispecific antibodies, these concerns need to be addressed in more detail.

## 7. Perspective

There is a growing interest in the application of IgA antibodies for clinical use. Pre-clinical work demonstrates the potential of IgA antibodies as anti-cancer therapeutics. Yet, the next step to the clinic has not been made due to technical limitations in IgA half-life and antibody production. By sophisticated engineering strategies of IgA, many of the IgA-associated restrictions have been tackled. The developability of IgA antibodies has been greatly improved, allowing for production levels similar to IgG antibodies. Engineered IgA shows a less complicated glycan profile due to the substitution of *N*-glycosylation motifs, resulting in more homogeneity. However, some limitations still need improvement, such as increased serum half-life of IgA either by introducing FcRn-binding sites or by combining the advantages of IgG antibodies into an IgA molecule. In addition, different strategies engaging neutrophils using bispecific molecules show promise as well, yet also raise some major concerns that need to be addressed to progress as therapeutic agents.

To summarize, in order to advance IgA antibodies as therapeutic agents, engineering is undoubtedly required. The examples described all have their pros and cons, which, by combining one or more strategies, can be refined. Although a long serum half-life would be desirable in many instances, a half-life of 6 days in man is more than most small molecules or scFv-based fragments accomplish. Acting in the spectrum between prolonged IgG exposure and short-lived small molecule therapeutics will open up new avenues for IgA therapy, providing a short but powerful blow to tumors.
